# Genomic Newborn Screening for Pediatric Cancer Predisposition Syndromes: A Holistic Approach

**DOI:** 10.3390/cancers16112017

**Published:** 2024-05-26

**Authors:** BalaSubramani Gattu Linga, Sawsan G. A. A. Mohammed, Thomas Farrell, Hilal Al Rifai, Nader Al-Dewik, M. Walid Qoronfleh

**Affiliations:** 1 Department of Research, Women’s Wellness and Research Center, Hamad Medical Corporation (HMC), P.O. Box 3050, Doha 0974, Qatar; 2 Translational and Precision Medicine Research, Women’s Wellness and Research Center (WWRC), Hamad Medical Corporation (HMC), Doha 0974, Qatar; 3 QU Health, College of Medicine, Qatar University, Doha 0974, Qatar; 4 Neonatal Intensive Care Unit (NICU), Newborn Screening Unit, Department of Pediatrics and Neonatology, Women’s Wellness and Research Center (WWRC), Hamad Medical Corporation (HMC), Doha 0974, Qatar; 5 Genomics and Precision Medicine (GPM), College of Health & Life Science (CHLS), Hamad Bin Khalifa University (HBKU), Doha 0974, Qatar; 6 Faculty of Health and Social Care Sciences, Kingston University and St George’s University of London, Kingston upon Thames, Surrey, London KT1 2EE, UK; 7 Healthcare Research & Policy Division, Q3 Research Institute (QRI), Ann Arbor, MI 48197, USA

**Keywords:** newborn screening, cancer predisposition syndromes, gene panel, pediatric cancer, genetic predisposition, cancer genetics, NGS

## Abstract

**Simple Summary:**

Overall, this review offers a comprehensive and insightful exploration of the significance, implementation, and challenges of genomic newborn screening for pediatric cancer predisposition syndromes (CPSs). It emphasizes the importance of next-generation sequencing (NGS) in uncovering germline mutations that are responsible for CPSs in childhood malignancies. It delves into the selection criteria for screening, ethical considerations, gene panel selection, and the integration of established and emerging genes in CPS into large-scale newborn screening programs in healthcare systems. It also stresses the importance of early detection and its potential impact on pediatric care and outcomes, thus providing valuable information for healthcare professionals, researchers, and policymakers in the field of pediatric oncology and genetics.

**Abstract:**

As next-generation sequencing (NGS) has become more widely used, germline and rare genetic variations responsible for inherited illnesses, including cancer predisposition syndromes (CPSs) that account for up to 10% of childhood malignancies, have been found. The CPSs are a group of germline genetic disorders that have been identified as risk factors for pediatric cancer development. Excluding a few “classic” CPSs, there is no agreement regarding when and how to conduct germline genetic diagnostic studies in children with cancer due to the constant evolution of knowledge in NGS technologies. Various clinical screening tools have been suggested to aid in the identification of individuals who are at greater risk, using diverse strategies and with varied outcomes. We present here an overview of the primary clinical and molecular characteristics of various CPSs and summarize the existing clinical genomics data on the prevalence of CPSs in pediatric cancer patients. Additionally, we discuss several ethical issues, challenges, limitations, cost-effectiveness, and integration of genomic newborn screening for CPSs into a healthcare system. Furthermore, we assess the effectiveness of commonly utilized decision-support tools in identifying patients who may benefit from genetic counseling and/or direct genetic testing. This investigation highlights a tailored and systematic approach utilizing medical newborn screening tools such as the genome sequencing of high-risk newborns for CPSs, which could be a practical and cost-effective strategy in pediatric cancer care.

## 1. Introduction

Population-based newborn screening/testing is an essential global healthcare initiative to identify neonates that have a high risk of childhood-onset cancers. Pediatric cancers are heterogeneous in nature and broadly categorized into central nervous system (CNS) tumors, non-CNS solid tumors, and hematological malignancies [1]. Although the cause of pediatric malignancies is not well understood and is typically regarded as idiopathic, recent advancements in the genomic field have provided better knowledge on the role of inherited and germline genetic factors in pediatric cancers. Several germline mutations in pediatric cancer patients have been identified [2,3,4]. Using the genomic newborn screening method, it is highly possible to diagnose conditions for which effective therapy and medications are available so that treatment can be given early enough to ameliorate congenital diseases. Childhood cancers constitute less than 1% of all new cancer diagnoses each year. While most of the research has centered around adult cancer populations, a particular study on pediatric cancers revealed that over 25% of patients seen ought to potentially be referred to a cancer genetics clinic based on various factors such as family history, tumor type, or physical findings [5]. Failure to identify these patients using cancer surveillance programs could result in a delay in assessing individuals with cancer predisposition syndromes (CPSs), potentially hindering the early diagnosis of pediatric cancer [6]. Diverse clinical screening tools have been suggested to identify patients with an increased risk of developing CPS [7,8,9,10]; however, their efficacy has varied across study populations, and their strategies have been inconsistent [11,12,13]. Despite the availability of cancer surveillance guidelines for early detection in high-risk infants and children, there are a limited number of research studies showing accurate clinical diagnosis of pediatric CPSs using newborn screening methods. Evidence from pediatric research studies shows that cancer surveillance and diagnosis of high-risk infants and children carrying a germline cancer-predisposing mutation using newborn screening methods may result in improved outcomes [14].

Newborn genome sequencing could be efficiently used to identify newborns who are at risk of developing cancer in childhood that is now not detectable by standard newborn screening methods. This review elaborates on significant steps involved in genomic newborn screening and integrating it into clinical settings for the screening of CPSs in newborns to diagnose pediatric cancer at an early stage, thus setting a stage for future research in newborn genome sequencing in CPSs.

### 1.1. Background and Rationale—Newborn Screening

Traditional newborn screening (tNBS) is a crucial global public health policy allowing early detection of disorders that would cause irreversible clinical damage if not recognized at birth [15]. Newborn screening (NBS) is a mandatory part of pediatric care, describing a set of laboratory tests, including metabolic, hematologic, and endocrinologic tests, as well as genetic analyses. The initial screening is followed by confirmatory diagnostic testing on individuals who were found to be at risk [16]. NBS is a World Health Organization (WHO)-approved pediatric strategy implemented across many countries. The strategy of early detection of immediately treatable disorders has enhanced not only the quality of life of newborns but also, in many cases, saved their lives [17].

The tNBS programs initially screened for PKU (phenylketonuria), a genetic disorder that can lead to irreversible brain damage without immediate action [18]. The detection of PKU through NBS has proven to be highly effective in facilitating early intervention, thereby mitigating potential complications for the inflicted child. This accomplishment sparked global conversations regarding the possibility of conducting screenings for additional disorders in newborns at the time of birth [19]. The WHO published Wilson and Jungner’s Principles in 1968 as a justification for a condition’s exclusion or inclusion in a screening program. Numerous factors are taken into consideration when evaluating a test, including its analytical validity, clinical utility, and accessibility to treatment or therapy [20]. Wilson and Jungner’s Principles were adopted by NBS programs, which then broadened their scope to encompass additional conditions. Over the years, the screening program expanded to include more disorders. In 1974, congenital hypothyroidism was added, and later on, other disorders were included as well [19]. However, identifying the condition at an early stage could significantly reduce the ‘diagnostic odyssey’ experienced by both patients and parents [21]. Globally, there is substantial variation in the extent and coverage of screening in public health newborn screening programs. As an example, the United Kingdom (UK) conducts screenings for nine conditions, while the United States (US) has an average of 35 screenings and Australia has 25 screenings [22,23]. Moreover, the countries in the European Union (EU) lack concordance in total number of specific conditions to be screened in newborns using genomic NBS, whereas the Middle East and North Africa (MENA) have established traditional NBS programs for few genetic and metabolic conditions [24,25]. Each nation may have a specific list of disorders included in its NBS program, depending on factors like healthcare priorities, resources, and the prevalence of certain conditions within the population [26].

In 2006, the American College of Medical Genetics and Genomics (ACMG) created the Recommended Uniform Screening Panel (RUSP), which details the standardized list of disorders/conditions in NBS programs across the States in the US [27]. For over sixty years, biochemical screening (traditional newborn screening—tNBS) has demonstrated its effectiveness in identifying and addressing treatable disorders in newborns. This screening helps to prevent or greatly improve the negative outcomes associated with these genetic conditions [19]. Presently, tNBS incorporates disease-specific analytes, which reduce the incidence and mortality rate of numerous severe metabolic and genetic disorders that manifest in children at an early age. This includes the likes of classical homocystinuria, cystic fibrosis, phenylketonuria, and hemoglobinopathies [28,29,30,31,32,33].

Overall, the extent and coverage of newborn screening programs can vary significantly due to a range of factors. Efforts to standardize and improve newborn screening practices can help ensure that all infants have access to timely and effective screening services. Additionally, the advancement of technology and healthcare systems may lead to expansions or enhancements of NBS programs over time.

### 1.2. Qatar Newborn Screening Program

Qatar Expanded Newborn Screening Programme (QENSP) has conducted screening for metabolic and endocrine problems in over 400,000 babies since its inception in 2003. The screening process is typically conducted for over 98% of all births. Many of these screened disorders can lead to growth failure, delayed learning, and lifelong impairments. The newborn screening test panel consists of 83 disorders, which include the following: Galactosemia, Biotinidase Deficiency, congenital hypothyroidism, Congenital Adrenal Hyperplasia, 25 Amino Acid Disorders, 16 Fatty Acid Oxidation Disorders, 25 Organic Acidemias, and 12 hemoglobinopathies. Currently, Guanidinoacetate Methyltransferase Deficiency, Severe Combined Immunodeficiency, and four Lysosomal Storage Diseases are being validated. These screens are conducted utilizing a diverse array of techniques like photometric, liquid chromatography, and liquid chromatography–tandem mass spectrometry methods [34].

Genetic diseases exemplify a considerable source of morbidity and mortality in populations with high rates of consanguinity, like the Arab population. They are projected to be the second leading cause of infant mortality in the State of Qatar. Most of the genetic disorders that plague the Arab population are dissimilar to other populations; hence, genetic tests developed for other populations are of very limited value for the Arab communities. In conjunction with Qatari-specific genetic variants disclosed by next-generation sequencing (NGS), stakeholders from Qatar have compiled a list of unique concerns that have been documented to cause hereditary diseases within the population [35,36].

More than 3500 rare genetic diseases, along with germline pediatric cancers, are known to manifest symptoms within a few years of childhood. Generally, the diagnostic process for the detection of rare genetic diseases tends to be time-consuming, so the diagnostic odysseys can be overcome by introducing rapid genomic newborn screening methods. The genome sequencing of newborns can provide complete genetic information on the entire genomic DNA, and it can be a cost-effective test that can support the diagnosis of most genetic diseases, which enables clinicians to rapidly determine the cause of rare genetic diseases in newborns [35,36].

A pilot study from Qatar, where the First Q-Chip (Qatar-microarray gene Chip)-Based Expanded Genomic Newborn Screening tool was developed to screen the spectrum of genetically treatable diseases for newborns. The Q-chip will be used on umbilical cord blood post-delivery, which contains thousands of pathogenic genetic variants that are common in the Qatari population. The Q-Chip design and development was a consequence of the collaborative work led by the Qatar Genome Project that involved major local stakeholders [35,37]. There are ongoing efforts to implement genomic screening for newborns as a part of a precision medicine initiative in Qatar to improve the population’s health. Recently, Qatari newborn screening translational research initiatives were launched to utilize cord blood and dried blood spots to develop new screening methodologies (unpublished data).

### 1.3. Newborn Screening Using Genomic Technologies

After the Human Genome Project (HGP) concluded in 2001, excitement grew about the potential of genomics to improve our knowledge of diseases and to facilitate the development of novel diagnostics and therapies [38]. In the study undertaken by Chen et al., the researchers recruited approximately 30,000 neonates to undergo standard newborn screening and sequencing of a panel of genes for the detection of 128 diseases [39]. A total of 59 instances yielded false negative results in traditional newborn screening but were accurately detected with a focused newborn screening utilizing genomic technology. In addition, newborn genomic screening extends beyond traditional biochemical screening and encompasses a wider range of disorders. This method accurately identifies cases of disease by reducing false positives resulting from various circumstances. Research investigations on inborn errors of metabolism indicate that conventional newborn screening yielded inaccurate false positive results, which were subsequently identified using genomic analysis [40]. Hence, the focused analysis of babies’ complete range of genetic disorders using genome sequencing offers accurate detection capabilities in comparison to conventional newborn screening. This implies that the genomic sequencing of newborns could be deemed a suitable approach for first screening [28]. Genome sequencing has, thus, long been predicted to become part of NBS [41]. Several research programs, such as the BabySeq Project and GUARDIAN (Genomic Uniform-screening Against Rare Diseases in All Newborns), have focused on screening asymptomatic newborns for around 160 disorders that can be treated [42,43].

As the field of genomic medicine progresses beyond current limits, it is now feasible to use sophisticated genomic tools to boost traditional newborn screening to detect hidden genetic disorders and cancer predisposition syndromes that go unnoticed by the current newborn screening. Newborn screening using genomic technologies such as NGS offers the potential for the efficient screening of newborns for more disorders at a lower overall cost per disease.

Several efforts to apply NGS to newborn screening have been underway for quite a few years [42,44,45,46,47,48], using gene panels, whole exome sequencing (WES), or whole genome sequencing (WGS) methodologies [49]. Targeted gene panels can be customized [“on-demand” design] for newborn screening to enrich the sequencing library with specific (targeted) gene regions to be read [50,51]. Achieving a good read depth and high coverage of medium- and large-sized gene panels permits the identification of significant small nucleotide polymorphisms (SNPs), small indels, and copy number variants (CNVs) overlapping the gene panel of interest. Genetic variants (SNP, CNV) identified in a CPS condition need to be confirmed via orthogonal assays. This review is a starting point for incorporating CPS panels in the genomic newborn screening (gNBS) screening program. We are aware that post-transcriptional events and epigenetic influences need to be considered; notwithstanding, the focus here is on early detection utilizing NGS technologies.

Integrating genomic sequencing into large-scale newborn screening programs can greatly enhance the early detection of rare diseases that can be treated. Also, the storage of genomic data could provide long-term health benefits and support future research endeavors as well as healthcare policies [52].

### 1.4. Pediatric Cancers—Global Incidence, Mortality, and Survival Rate

Pediatric cancer, despite its low overall incidence rate, is the leading non-injury-related cause of death amongst children aged 0–19 years in the USA [53]. During the past decade, every year, approximately 0.3 million children have been diagnosed with cancer worldwide [54,55]. The most commonly diagnosed cancers were leukemias, lymphomas, neuroblastomas, and brain and CNS tumors among children aged 0–14 years [54]. On the contrary, the most common cancers identified in the adolescent age group were lymphomas, melanomas, epithelial tumors, leukemias, germ cell tumors, and sarcomas [54]. During 2001–2016, the highest mortality rate observed in pediatric cancers belongs to leukemias (28.5%), brain and other nervous systems (26.9%), and bones and joint tumors (9%) [56,57]. The overall mortality rate of pediatric cancers among children and adolescent age groups was approximately 25 per million in the USA [56,57]. During 2016–2022, the mortality rate decreased by 1.5% upon the availability of advanced treatment and supportive care for pediatric leukemia and lymphoma [58]. However, the mortality rate of soft-tissue, brain, and bone cancers remained unchanged. In 2011, brain tumors were considered the leading cause of tumor death besides leukemia [57].

### 1.5. Genomic Medicine and Pediatric Cancer Predisposition Syndromes (CPSs)

Pediatric CPSs are a group of genetic disorders characterized by an augmented risk of developing pediatric cancers. CPSs are commonly caused by inherited genetic mutations in specific genes, which are implicated in DNA repair mechanisms, cell cycle regulation, or tumor suppression pathways. Some typical examples include neurofibromatosis type 1, Li–Fraumeni syndrome, and familial adenomatous polyposis [59]. However, recent genomic studies show that 10% of pediatric cancer patients have an underlying CPS [60], and a significant proportion of pediatric cancers are associated with germline mutation in cancer predisposition genes [3,61]. Research studies imply that the prevalence of germline mutation is high in children detected with choroid plexus carcinoma (*TP53*) and malignant rhabdoid tumors (*SMARCB1*). The identification of inherited genetic variants in neonates will shorten the diagnostic odyssey and allow clinicians to deliver genetic counseling recommendations to patients and families. Pediatric cancer’s increased risk can now be ascribed to the full spectrum of germline genetic changes, including single-site mutations, translocations, inversion, trisomies, and genome imprinting [62]. Missense, nonsense, splice site, and silent mutations confer varying risks of disease development based on both the genomic location of the genetic change and the cellular function of the affected gene. High-frequency CNVs were reported in the genome of patients affected by Li–Fraumeni syndrome [63]. Additionally, ~40% of the cancer genes identified in the “Census of Human Cancer Genes” were influenced by CNVs [62,64].

More than 100 cancer-predisposing genes have been discovered that are believed to play an important role in cancer phenotype [65,66]. The identification of pathogenic genetic variations in genes that are linked to a higher probability of early childhood tumors could be integrated into expanded newborn screening programs. The identification of cancer-causing genetic variations would trigger the application of established clinical care recommendations currently employed by pediatric oncologists for infants and children with known CPS [66]. It is well-reported that newborn testing and surveillance would reduce cancer deaths by ~7.8% [66]. In addition, the early identification of these syndromes through genomic newborn screening—gNBS enables proactive surveillance and interventions to mitigate cancer risk.

### 1.6. Pediatric Cancers—Importance of Early Detection

The early detection of cancer leads to effective care that results in better survival and less intensive and suffering treatments [67]. Delay in cancer diagnosis leads to difficulty in having a correct diagnosis due to various complications, and patients may not receive effective treatment. The WHO has described two early detection approaches: (i) early diagnosis, the recognition of symptomatic cancer in patients; (ii) screening, the identification of asymptomatic disease in a healthy target population [68,69]. In general, it is not practical to screen for cancers in children since the cause of most is unknown [69]. Only a few cancers are known to be caused by inherited genetic mutations, environmental factors, or chronic infectious agents like HIV and hepatitis B [70,71].

The CPS combined has contributed <10% of all childhood cancer cases [3,72,73,74,75,76]; however, different cancer types have specific molecular pathways and biological mechanisms that are common in nature [77], and it plays an essential role in a better understanding of cancer development. CPS is a germline inherited disorder in which there is a moderate to high risk of certain types of cancer. Identifying CPSs in neonates is significant, as it enables surveillance at the genetic level and the early detection of pediatric cancers. Diagnosis of a CPS in newborns is pursued to warrant personalized therapy for the diagnosed condition, diminish treatment toxicities, and provide proper clinical follow-up along with genetic counseling for the family. Even though modification in diagnostic testing and treatment is needed in some CPSs, the screening and diagnosis of CPSs in children with cancer is largely predicated on clinical suspicion and family history to initiate prompt referral to a clinical geneticist.

The early detection of pediatric CPS has several potential benefits. It allows for the initiation of surveillance programs, which can lead to the pre-symptomatic detection of malignant neoplasms, thus improving treatment options and survival outcomes [78]. Dedicated surveillance has been shown to detect malignancies early and improve the overall survival of several CPSs [79]. Additionally, early detection enables tailored therapy and surveillance for the probands and their families, leading to better patient management [80].

### 1.7. Genomic Landscape of Pediatric Cancers

Recent advances in genome analyses have remarkably enhanced our understanding of elucidating the genetics or genomic landscape of several pediatric cancers [81]. Because of a better insight into the disease and treatment regimes, nearly 80% of affected children have been cured lately worldwide; still, cancer is considered the leading cause of death among children over one year of age, depending on its type, etiology, and other factors [82]. Genome sequencing studies indicated that the genomic landscape of pediatric cancer is highly assorted and noticeably different from adult cancer in many cases.

**Figure 1:**
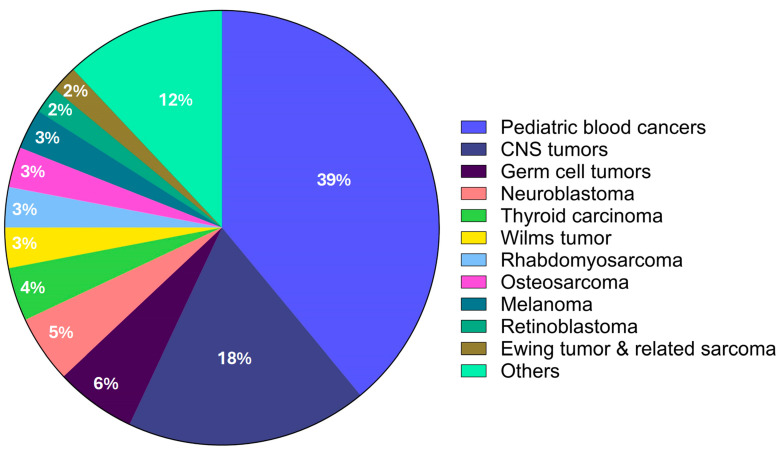
Incidence of pediatric cancer among individuals under the age of 19, as determined from data provided by the Centers for Disease Control and Prevention (CDC). (United States Cancer Statistics Data, https://wonder.cdc.gov/cancer.html) accessed on 15 March 2024 [86].

To advance the development of targeted and less harmful treatments, it is essential to fully understand the entire genetic makeup of pediatric malignancies. These malignancies differ from adult tumor malignancies in terms of their histological examination and molecular subtypes. In recent years, several sequencing projects have been initiated to study specific entities. However, there has been limited research on pediatric pan-cancer that has only examined mutation rates, germline predisposition, and changes in epigenetic regulators [3,83,84]. One study found that 61% of children and young adults diagnosed with solid tumors possessed germline pathogenic genetic variants in a wide range of candidate genes that were not known to be cancer-predisposing genes [85]. Pediatric cancers caused by germline pathogenic variants are termed “familial cancers” (occurs when a genetic change that increases cancer risk runs in the family rather than in the general population) because of the high penetrance of genetic variants and their susceptibility towards malignant formation. The main pediatric cancers, which together account for nearly 34.8% of all childhood cancers, are represented graphically here. These include neuroblastoma, retinoblastoma, Wilms tumor, osteosarcoma, rhabdomyosarcoma, and Ewing sarcoma. The CNS tumors include medulloblastoma, ependymoma, and astrocytoma (Figure 1).

### 1.8. Autosomal Dominant Form of Cancer Predisposition Syndromes

Most of the single-gene CPSs are caused by genetic variation or mutations in tumor suppressor genes, and they are inherited via the autosomal dominant condition [72]. The gene has a 50% chance of being equally inherited from the maternal and paternal sides and to both daughters and sons. Because of its total penetrance, the genetic variant’s phenotypic expression may skip a generation. Approximately 80% of all discovered de novo germline point mutations in offspring occur on the paternal allele, and most studies have linked a high percentage of de novo mutations (DNMs) in the offspring to the paternal age [87,88,89]. To summarize, the rate of DNA replication and cell division in germline cells is significantly higher in males compared to females. Consequently, the probability of developing germline DNMs in spermatocytes and oocytes is proportional to the sex-specific rate of DNA replication and cell division. Thus, the majority of germline DNMs in the progeny are derived from the father, although their quantity rises with both the mother’s and father’s age at conception [90].

Hereditary retinoblastoma—*RB1*, a tumor suppressor gene, is the first gene linked to a childhood CPS [91], and those carrying a pathogenic variant in this gene go with a primarily increased risk for retinoblastoma (Table 1). Genetic variants in *RB1* have also been reported for increased risk for osteosarcoma and malignant melanoma in childhood, along with other tumors later in life [92]. In cases of hereditary retinoblastoma (RB) in children, the mutation is typically de novo. As a result, the parents are unaffected, and the chances of siblings being affected are very low. In the case of a child with RB, there is a 45% chance that their offspring will also develop the same tumor [93]. Up to 80% of all heritable retinoblastoma cases can be attributed to de novo germline mutations in the *RB1* gene [90,94]. It is crucial to distinguish DNMs from inherited pathogenic or suspected pathogenic variants to conduct testing on first-degree family members [1]. It is worth mentioning that NBS can be used to exclude a CPS, especially where there is a family history of a high-penetrance syndrome, such as heritable retinoblastoma.

Li–Fraumeni syndrome—The Li–Fraumeni syndrome (LFS) is a rare, inheritable familial CPS caused by a pathogenic variant in the *TP53* gene [95]. A proband with sarcoma diagnosed before the age of 45 who had a first-degree relative with any cancer under 45 and another first- or second-degree relative with either sarcoma at any age or any cancer under 45 was part of the “classic” LFS pedigree. LSF greatly increases the risk of developing various types of childhood or adolescent cancer, including breast cancer, CNS tumors, adrenocortical carcinomas, osteosarcomas, and soft-tissue sarcomas (Table 1). Additionally, it is linked with a higher risk of obtaining several types of additional cancer like leukemia, lymphoma, gastrointestinal cancers, and cancers of the head and neck, kidney, larynx, lung, skin, ovary, pancreas, prostate, testis, and thyroid. The risk of developing cancer in individuals with LFS or *TP53* pathogenic variants is very high, estimated to be 50% by age 40 years and up to 90% by age 60 at ≥70% for men and ≥90% for women during their lifetime [96,97]. It has been well reported that approximately 50% of *TP53* variants that are predicted to be pathogenic in osteosarcoma patients are de novo [98], whereas 14% of de novo *TP53* variants contribute to LFS during embryonic development [99].

**Table 1:** Genes involved in germline cancer predisposition syndromes of various cancer types (adapted from Pizzo and Poplack’s *Pediatric Oncology*, *8th edition* [77,100,101]).

Syndrome (Mode of Inheritance)	Gene(s)	Tumor Type
Ataxia-telangiectasia (AR)	*ATM*	leukemia, lymphoma
Basal cell nevus syndrome (Gorlin syndrome) (AD)	*PTCH*, *SUFU*	medulloblastoma
Beckwith–Wiedemann syndrome (AD)	*CDKN1C*, *H19*, *IGF2*, *KNBQOT1*	Wilms tumor, neuroblastoma, hepatoblastoma, rhabdomyosarcoma
Birt–Hogg–Dubé syndrome (AD)	*FLCN*	renal cell tumors
Bloom syndrome (AR)	*BLM*, *RecQL3*	oropharyngeal carcinoma, breast, colon carcinoma, osteosarcoma, leukemia, lymphoma, melanoma
Bohring–Opitz syndrome (AD)	*ASXL1*	medulloblastoma, Wilms tumor
Congenital central hypoventilation Syndrome (AD)	*PHOX2B*	neuroblastoma, ganglioneuroma, ganglioneuroblastoma
Constitutional mismatch repair deficiency (CMMRD) syndrome (AR)	*MLH1*, *MSH2*, *MSH6*, *PMS2*	high-grade glioma, medulloblastoma
Costello syndrome (AD)	*HRAS*	neuroblastoma, bladder carcinoma, rhabdomyosarcoma
Cowden syndrome (AD)	*PTEN*	breast carcinoma, thyroid carcinoma, renal cancer, colorectal carcinoma, melanoma, endometrial tumors
Dyskeratosis congenita (AD)	*DKC1*, *TERC*, *TERT*	squamous cell carcinoma, gastric carcinoma, myelodysplasia, leukemia
Dysplastic nevus syndrome (AD)	*CDKN2A* and others	melanoma
Fanconi anemia (AR)	*FANCA*, *FANCC*, *FANCG*	leukemia, hepatocellular, esophagus, head and neck, cervix, Wilms tumor, medulloblastoma, neuroblastoma, embryonal tumors
Familial acute myeloid leukemia (AD)	*RUNX1*	leukemia
Familial adenomatous polyposis (AD)	*APC*	hepatoblastoma, medulloblastoma, papillary thyroid carcinoma, intestinal carcinoma, pancreatic adenocarcinoma, desmoid tumors
Familial paraganglioma/pheochromocytoma (AD)	*SDHB*, *SDHAF2*, *SDHC*, *SDHD*	paraganglioma, pheochromocytoma, gastrointestinal stromal tumor
Hereditary pleuropulmonary blastoma (AD)	*DICER1*	pineoblastoma, meduloepithelioma, thyroid, nasal chondromesencymal hamartoma, pleuropulmonary blastoma, cystic nephroma, renal sarcoma, Wilms tumor, mesenchymal hamartoma, sertoli-leydig cell tumor, rhabdomyosarcoma
Hereditary breast/ovarian cancer (AD)	*BRCA1*, *BRCA2*	breast, ovarian, prostate, pancreatic
Hereditary non-polyposis colon cancer (Lynch syndrome) (AD)	*MLH1*, *MSH2*, *PMS2*, *MSH6*	colon, uterine, gastric, endometrial, small bowel, sebaceous gland
Hyper parathyroid-Jaw tumor (AD)	*CDC73*	parathyroid cancer, jaw ossifying fibroma, Wilms tumor, ovarian carcinoma
Li Fraumeni syndrome (AD)	*TP53*, *CHEK2*	leukemia, melanoma, glioma, choroid plexus carcinoma, breast carcinoma, Wilms tumor, adrenal carcinoma, osteosarcoma, soft tissue sarcomas
Multiple endocrine neoplasia type 1 (AD)	*MEN1*	parathyroid, pancreas, gastrinomas, insulinoma, carcinoid tumors
Multiple endocrine neoplasia types 2A (AD)	*RET*	thyroid medulla, pheochromocytoma
Mulibrey nanism (AR)	*TRIM37*	thyroid carcinoma, Wilms tumor, renal papillary carcinoma, pheochromocytoma, ovarian carcinoma, endometrial adenocarcinoma
Neurofibromatosis type 1 (AD)	*NF1*	gliomas, gastrointestinal stromal tumor, dermal neurofibroma, malignant peripheral nerve sheath tumor, juvenile myelomonocytic leukemia
Neurofibromatosis type 2 (AD)	*NF2*	schwannomas, meningioma, ependymoma, retinal hamartoma
Noonan syndrome (AD)	*PTPN11*, *SOS1*, *RAF1*, *KRAS*	dysembryoplastic neuroepithelial, neuroblastoma, leukemia, juvenile myelomonocytic leukemia
Nijmegen breakage syndrome (AR)	*NBS1*	lymphoma, leukemia
Peutz–Jeghers syndrome (AD)	*LKB1*	breast carcinoma, lung carcinoma, colon, pancreatic adenocarcinoma, ovarian carcinoma, sertoli cell tumor
Proteus syndrome (AD)	*AKT1*	parotid adenoma, ovarian cystadenoma
Simpson–Golabi–Behmel syndrome (XLR)	*GPC3* or *GPC4*	medulloblastoma, Wilms tumor, neuroblastoma, hepatoblastoma
Tuberous Sclerosis (AD)	*TSC1*, *TSC2*	subependymal giant cell astrocytoma, Angiomyolipoma, renal cell carcinoma
Retinoblastoma (RB) (AD)	*RB1*	retinoblastoma, osteosarcoma, melanoma, pinealoblastoma, lung carcinoma
Von Hippel–Lindau syndrome (AD)	*VHL*	renal cell carcinoma, pancreatic islet cell tumors, pheochromocytoma
Werner syndrome (AR)	*WRN*	leukemia, melanoma, osteosarcoma, thyroid
WAGR syndrome (AD)	*WT1*	Wilms tumor
Weaver syndrome (AD)	*EZH2*	neuroblastoma
Wiskott–Aldrich syndrome (XLR)	*WASP*	leukemia, lymphoma
Xeroderma pigmentosum (AR)	*DDB2*, *ERCC1*, *ERCC2*, *ERCC3*, *ERCC4*, *ERCC5*, *POLH*, *XPA*, *XPC*	basal cell and squamous cell skin cancers, melanoma, stomach, leukemia

Abbreviations: LFS = Li–Fraumeni syndrome; MPNST = malignant peripheral nerve sheath tumor; RB = retinoblastoma; WAGR = WT, aniridia, genital abnormalities, and mental retardation; WT = Wilms’ tumor; AD = autosomal dominant; AR = autosomal recessive; XLR = X-linked recessive.

Constitutional mismatch repair deficiency syndrome (CMMRD)—CMMRD is a rare and autosomal recessive CPS caused by pathogenic biallelic variants in *MLH1*, *MSH2*, *MLH1*, and *PMS2*. Biallelic variants in *MLH1* or *MSH2* variants resulted in an earlier onset of malignancy than *PMS2* or *MSH6* variants [100]. The main hallmark of CMMRD is multiple tumor development in brain, gastrointestinal, and hematological tumors, developing in the first and second decade of life [101] (Table 1).

Familial neuroblastoma—Neuroblastoma (NB) is the most commonly occurring malignant extracranial solid childhood tumor and accounts for nearly 15% of all cancer-related pediatric cancer mortality between the ages of 1 and 5 years [102,103,104]. It is defined as an embryonal neuroendocrine tumor arising from neural crest progenitor cells [104]. Due to the high variability in its presentation, NB exhibits unique clinical features, including a tendency for spontaneous regression of tumors in infants and a high frequency of metastatic disease at diagnosis in patients aged over 18 months [105] (Table 1).

Leukemia—Leukemia is one of the most common types of cancer in children, accounting for nearly 30% of all childhood cancers [106]. The majority of leukemia cases are developed without any known predisposing factors; however, it is well known that certain genetic and acquired germline mutations, bone marrow failure disorders, DNA repair defects, and constitutional chromosomal abnormalities are associated with an increased risk of leukemia incidence [107]. Moreover, in a few cases, family pedigree has exhibited a high risk of developing leukemia in the absence of known inherited mutations [108]. So far, three genes (*CEPBA*, *RUNX1*, and *GATA2*) have been discovered that can be passed down in an autosomal dominant manner, increasing the likelihood of developing leukemia (Table 1).

Familial adenomatous polyposis (FAP)—FAP is an autosomal dominant condition due to a mutation in *APC* (adenomatous polyposis coli) gene on chromosome 5q22. It has a reported incidence of 1 in 8000 in the Western population, and it is quite common in the Ashkenazi Jewish population [109]. Epigenetic modification in the APC promoter 1B region and germline inherited mutation (p.I1307K) in the *APC* gene has almost 100% penetrance, which leads to colonic polyposis [110]. In contrast, 20% of the patients with classic FAP may test negative due to de novo mutation and somatic mosaicism. It is well reported that up to 25% of FAP cases exhibit de novo *APC* mutations [111]. In some de novo cases, somatic mosaicism caused by an *APC* mutation has been identified as the genetic cause of a FAP/attenuated FAP (AFAP) phenotype, which is likely to be underdiagnosed entity [112]. This phenomenon is likely to be underestimated and not fully diagnosed. When encountering unexplained adenomatous polyposis, it is important to take into account the possibility of somatic mosaicism in the *APC* gene [112]. Children with an inherited FAP condition tend to develop benign polyps as early as their teenage years [113]. The common symptoms of FAP are blood or mucus in the stools or bleeding in the rectum, diarrhea or constipation, palpable abdominal masses, and weight loss [114] (Table 1).

Neurofibromatosis type 1—Neurofibromatosis type 1 (NF1), a.k.a. von Recklinghausen syndrome, is one of the prevalent genetic neurocutaneous disorders caused by the mutation of a tumor suppressor gene, Neurofibromin 1 [115], which inhibits the Ras protein [77]. Neurofibromatosis type 1 is associated with a mutation in the large NF1 gene. As a variant in a tumor-suppressing gene, it seems to follow the two-hit theory, as tumors associated with *NF1* show a loss of heterozygosity [116,117]. NF-1 is manifested by developmental changes in the nervous system, bones, and skin in one in every 3000 births [115,118]. Individuals affected by *NF1* have a high predisposition to develop varying numbers of neurofibromas, gliomas of the optic tract, other low-grade gliomas, and pheochromocytomas exhibit a benign course; however, they are at risk of developing malignancy or tend to develop into malignant tumors [119]. Optic gliomas and malignant peripheral nerve sheath tumors (MPNSTs) are the most frequently observed types of cancer in children with NF1 [116,120], with a high incidence of acute myeloid leukemia (AML) [121]. Moreover, the diagnosis or identification of individuals with a genetic predisposition to develop malignant tumors can result in a change of treatment plan, specific follow-up of adverse treatment effects, and early detection of second neoplasia [122]. Early detection of pediatric cancer predisposition has the potential to improve outcomes and reduce mortality rates through targeted surveillance and personalized treatment approaches (Table 1).

### 1.9. Aim of Pediatric CPS Screening Program

The screening program for pediatric CPSs aims to identify children who are at increased risk of developing malignancies due to cancer predisposition syndromes. The screening protocols involve regular surveillance, including imaging, to detect tumors or genetic variants in asymptomatic children [78]. The goal is to detect malignant neoplasms before symptoms appear to improve treatment options and survival outcomes [123]. The screening program also aims to minimize false-negative and manage false-positive results of imaging tests, such as whole-body MRI [13]. Standardized nomenclature is important for reporting risk stratification and guiding patient management [124]. Timely communication of results is crucial to alleviate anxiety. The newborn screening program for CPSs is multidisciplinary and longitudinal, requiring the development of regularized frameworks to enhance diagnostic performance and improve the patient experience.

## 2. Overview of Pediatric Cancer Predisposition Syndromes

### 2.1. Common Genetic Syndromes Associated with Pediatric Cancer

The most common pediatric CPSs include neuroblastoma (familial), retinoblastoma (hereditary), and Fanconi anemia [125]. Other common syndromes include Ataxia-telangiectasia, Beckwith–Weidemann syndrome, Bloom syndrome, DICER1 tumor predisposition syndrome, Dyskeratosis congenita, and Juvenile polyposis [126]. These syndromes are responsible for a higher risk of developing various types of pediatric cancers and are often caused by germline mutations in tumor suppressor genes [127]. Genetic testing and evaluation are important for diagnosing these syndromes, as they have implications for patient management, surveillance, and risk-reducing interventions [128]. Common germline pediatric cancer predisposition and pediatric cancer-related syndromes are summarized in Table 1. Even though many of them are usually manifest in adult age, they may also cause pediatric cancers.

CPSs account for around 10% of pediatric cancer cases [3,72,73,74,75,76]. They exhibit distinct molecular pathways and genetic mechanisms that are shared with various other types of cancer [77]. Additionally, CPSs play a crucial role in advancing our understanding of pediatric cancer development. The identification of these syndromes can also impact treatment decisions, such as the use of targeted therapeutics or avoidance of radiation. Therefore, it is recommended to screen pediatric oncology patients for germline mutations to identify cancer predisposition syndromes and provide appropriate care.

### 2.2. Impact on Health and Development

CPSs have significant health and developmental impacts. The presence of CPS can lead to changes in cancer treatment regimen and the need for surveillance due to an increased risk of developing additional primary malignancies [129,130]. Newborns may inherit the CPS germline, highlighting the importance of genetic testing and counseling for potential carriers [129,130]. Additionally, CPSs can encompass non-cancer-related problems, such as behavioral or immunologic disorders, that require specific attention and care [129]. The complex clinical management of CPSs necessitates a multidisciplinary team with specific training. Overall, the CPS diagnosis has broad implications for patients and their families, including targeted treatment strategies, surveillance, risk-reducing interventions, and reproductive options. Pediatric urologists play a crucial role in identifying genitourinary (GU) manifestations of CPSs, which can prompt genetic testing and impact treatment decisions.

### 2.3. Genetic Basis and Inheritance Patterns of Pediatric CPS

Pediatric CPSs are associated with genetic risk factors. Germline alterations in genes, such as *RET*, RB1, TP53, DICER1, SUFU, PTCH1, SMARCB1, SMARCA4, *WT1*, APC, ALK, PHO2XB, *CDKN2A*, *NF1*, and *NF2*, have been identified as potential pathogenic variants in pediatric and young adult patients with sarcomas [131] (Table 1). These alterations confirm the diagnosis of cancer predisposition syndrome. More studies revealed that about 10% of pediatric oncology patients have an underlying CPS, most often arising from germline mutations in tumor suppressor genes [60]. The genetic risk factors for pediatric cancer predisposition syndromes are highly constrained, indicating selective mutational pressure on these genes [129]. It is well reported that 10–18% of pediatric cancer patients have an underlying genetic susceptibility to their disease, and with the advancement in genomic technologies, more genes and syndromes associated with cancer predisposition are being discovered [132].

For the past five decades, Wilson and Jungner’s principles (1968) guided decisions on including various inherited and genetic diseases in large-scale population-based screening programs. These guideline criteria can be well applied to determine CPS conditions in newborn genomic screening programs [133]. Advancements in newborn screening methods are more efficient in testing many metabolic conditions, which leads to extension in conditions screened over time. Despite these changes in NBS criteria, consent and privacy practices within demographic populations remain unchanged. The expansion of NBS programs to include genomic testing would allow for the detection of rare disease conditions that may be left undetected using traditional methods.

## 3. Screening Methodology

### 3.1. Selection Criteria for Screening

The selection criteria for screening pediatric CPSs is to evaluate the significance of genes in childhood cancer and the available evidence that supports gene variants in the development of cancer. Clinical criteria such as family and patient’s medical history, specific clinical signs, and specific histopathological tumor subtypes are also considered [134]. In addition, routine screening for germline mutations in pediatric neuro-oncology patients has been found to reveal unsuspected CPSs and impact care [128]. However, the efficacy of clinical checklists in reliably detecting genetic cancer predisposition in children with cancer is still insufficient, highlighting the need for routine germline sequencing of pediatric cancers [135]. Surveillance protocols for pediatric patients with CPSs often include regular imaging, such as whole-body MRI, to detect malignant neoplasms [78].

### 3.2. Ethical Considerations and Informed Consent

Current ethical and public policy issues associated with newborn screening practices apply to large-scale genome screening as well, and most of the ethical issues are exacerbated by the reason that the individual receives more information in large-scale genome-wide screening as compared to the conventional testing procedure. Population-based NBS is the most successful public health intervention, with the main focus on treating and preventing serious health conditions in newborns [136,137]. Currently, most neonates in developed and developing countries undergo NBS tests to diagnose early-onset diseases [138]. Introducing genome sequencing for newborn screening of CPS brings better opportunities to identify more infants who are predisposed to germline genetic variants associated with CPS, and early interventions can bring effective treatment outcomes for pediatric cancers. Moreover, more precautions should be taken to ensure that newborn genomic screening of CPS is utilized in a manner that does not compromise the results interpretation or societal support of existing NBS programs.

One of the main issues involved in population-based screening of newborns is whether parental consent is required before proceeding with newborn screening for pediatric cancers. The American Academy of Pediatrics (AAP) and the ACMG recommended mandatory offering of newborn screening for all neonates. In the case of a family history of inherited disorders, parents should be provided with knowledge and counseling on newborn screening results, parents can have the option of refusing further downstream procedures, and informed refusal should be taken into consideration [139]. For screening CPSs in newborns, it is mandatory to obtain authorization from parents or guardians for the predictive genetic testing of asymptomatic newborns at risk of pediatric cancers [139]. Moreover, maintaining efficient administration of population-based genomic screening programs for newborns in clinical settings is very important to prevent low participation rates. In Qatar, newborn genetic screening has already been implemented, as there are no ethical dilemmas, and its permissibility is relatively straightforward [140]. There is a significant risk of genetic disorders due to the prevalence of consanguineous marriages in several Muslim-majority countries in the region, including Qatar. Studies have shown that there is an increased prevalence of autosomal recessive genetic disorders such as cystic fibrosis, sickle cell anemia, and thalassemia, along with rare genetic disorders in Qatar [28,141,142]. There is a notable presence of genetic diseases such as glucose-6-phosphate dehydrogenase (G6PD), hemoglobinopathies, and other metabolic disorders in Saudi Arabia [143,144]. A study conducted on children in Saudi Arabia showed that around 40% of the children diagnosed with Hereditary Cancer Susceptibility Syndrome (HCSS) had a consanguinity rate of 90% [145]. To address these issues, Qatar has implemented mandatory screening for genetic disorders both before marriage and for newborns. An important ethical challenge arises from the possibility of discovering secondary findings that are unrelated to the primary purpose of the test. Newborn genomic screening programs in the MENA region are growing rapidly, especially in Qatar, despite political, ethical, and logistical challenges. In Qatar, screening is provided for all newborns of residents at no cost and >98% of the births are screened [52].

There are substantial research studies that provide compelling evidence for the clinical utility and cost-effectiveness of genomic sequencing of newborns for CPSs at an early point in the diagnostic trajectory, as pointed out above [6].

## 4. Panel of Genes for Screening

### 4.1. High-Risk Genes

Multiple reports indicated that approximately 10% of children with cancer possess a hereditary mutation in a gene that increases their susceptibility to developing cancer [14]. An American Association for Cancer Research (AACR) symposium in 2016 primarily focused on studying the 50 most common CPSs that increase the risk of developing cancer during the first two decades of life, which are classified into ten main groups: (1) Neurofibromatosis, (2) Li–Fraumeni syndrome, (3) Overgrowth syndromes and Wilms tumor, (4) Neural tumors predisposition, (5) Neuroendocrine syndromes, (6) Gastrointestinal cancer predisposition, (7) Leukemia predisposition, (8) DNA instability syndromes, (9) constitutional mismatch repair deficiency, and (10) other miscellaneous syndromes [14].

A community-based cross-sectional study was conducted (Mean age = 38.96 ± 14.16 years) on residents (women population) in the State of Qatar from April to December 2021 using a list of 64 single nucleotide polymorphisms (SNPs) associated with cancer predisposition syndrome. The results show that the genetic variant *MDM2*, G>T (rs2279744), was found significant with a *p*-value of less than 1 × 10^−5^. *MDM2* (Murine double minute 2) is widely known for its significant contributions to cancer development, including promoting sustained angiogenesis, metabolic reprogramming, growth stimulation, apoptosis evasion, metastasis, and immunosuppression. Genetic variation in *MDM2* promotes its increased expression levels, which leads to uncontrolled proliferation in many cancer types [146].

### 4.2. Comprehensive Gene Panel Selection

Since the advent of NGS, the diagnostic approach for identifying cancer predisposition variants has been shifting increasingly towards a genotype-first approach. To this date, only a few studies have combined comprehensive clinical data and WES/WGS sequencing in an unselected cohort of pediatric patients [12,147,148]. Gene panels are currently utilized globally to identify cancer predisposition alterations for hereditary cancers. These panels are available to adults who have a history of specific cancers, including breast cancer, ovarian cancer, skin cancer, and bowel cancer [149]. The gene panel used for hereditary cancers includes 20 genes that have been identified as having a predisposition for the development of cancers in early adulthood. These genes are *APC*, *BMPR1A*, *BRCA1*, *BRCA2*, *CDH1*, *CDK4*, *CDKN2A*, *HOXB13*, *MLH1*, *MSH2*, *MSH6*, *MUTYH*, *PALB2*, *PMS2*, *POLD1*, *POLE*, *PTEN*, *SMAD4*, *STK11*, and *TP53* [149].

Standard criteria and recommendations for the screening of widespread germline cancer-predisposing mutations in cancer-affected children are desperately needed. Consistent research outcomes on pediatric cancer genomics are being translated into routine clinical diagnostics [150]. The validated and reproducible pediatric CPS gene panel is inevitable for the successful diagnosis of germline genetic variations in genes that have a proven association with childhood cancer. Radboudumc (version DG2.17—232 genes) and STAGING study from Denmark (contains 314 genes) currently offer a well-established hereditary cancer gene panel, which is potentially related to childhood cancer predisposition [151]. Another study reported that two CPS gene panels, such as “Tumour predisposition—childhood onset (Version 2.1)” (114 genes) and “Childhood solid tumors cancer susceptibility (Version 1.6)” (83 genes) [151,152], are designed for pediatric cancer diagnosis. The gene lists for the above-mentioned panels were taken from recent literature sources where these genes were predominantly reported for newborn CPSs [3,65].

### 4.3. Integration of Established and Emerging Genes

Several recent studies have indicated that 60 genes have been associated with autosomal dominant CPSs [3]. There is a total of 633 non-silent germline variants identified in these genes. Out of these, 78 (12%) are considered pathogenic, 17 (3%) are possibly pathogenic, and 226 (36%) have uncertain significance. A total of 95 pathogenic variants were identified in 21 genes, which encompass 54 missense mutations, 14 nonsense mutations, 12 frameshift mutations, 9 splice-site mutations, and 1 in-frame deletion, as well as 5 copy-number alterations [3].

### 4.4. Considerations for Genetic Variants of Uncertain Significance (VUS)

One significant drawback of ordering excessively large gene panels is the notable increase in the number of variants of uncertain significance (VUSs) as more genes are assessed. According to recent studies, it has been found that over 44% of patients may receive one or more variants of uncertain significance, depending on the type of cancer and the specific test conducted [153]. Although some laboratories are actively sharing their data with ClinVar to encourage collaboration and address uncertain results, not all laboratories conducting cancer genetic testing are currently involved in this initiative. In some cases, insurance companies have pushed for increased data sharing and will only reimburse testing performed at labs that contribute to ClinVar [154].

Given the growing accessibility of extensive sequence data from diverse populations (such as ExAC and gnomAD) and the advancements in high-throughput functional assays, there is potential to better understand a larger number of VUSs. This reinterpreted data must be shared with clinicians and patients promptly, as it can provide valuable guidance for patient management [155,156,157]. It is crucial to provide updated interpretations, especially for minority patients who experience a higher occurrence of VUSs and may have limited knowledge about genomics, to help them grasp the complexities associated with a VUS [158,159,160,161]. Assessing the pathogenicity of a variant in hereditary cancer predisposition syndromes is particularly difficult due to the lack of a reliable clinical method. It can be challenging for a physician to identify VUSs, which makes decisions about medical therapy, genetic counseling, and the monitoring program more complicated [162]. However, it is important to counsel patients to adhere to guidelines based on their personal and/or family history, even in the absence of a confirmed mutation [161].

To address the questions regarding penetrance, cancer spectrum, and recommended clinical management, it is crucial to gather more data. This will allow for improved genotype–phenotype correlations, estimation of age- and gender-specific penetrance and expressivity, and identification of modifiable risk factors through enhanced data sharing. There are a few public registries, like ENIGMA (https://enigmaconsortium.org, accessed on 9 March 2024) and PROMPT (http://promptstudy.org), that provide support for these studies. However, it is important to note that the data available in these registries is only a tiny portion of the total number of mutation carriers identified through clinical means. It would be more efficient and effective if patients had the choice to provide masked clinical data to researchers. These data could be used to generate knowledge-based recommendations that have the potential to directly benefit patient care in the future.

### 4.5. Genomic Database for Childhood Cancers

Significant amounts of patient health data, known as big data, have been extensively utilized in adult oncology research to uncover new possibilities. Nevertheless, the utilization of big data is still in its early stages when it comes to pediatric cancer research. The US National Cancer Institute (NCI) has actively promoted the utilization of big data in various oncology subspecialties. As part of this effort, the NCI introduced the Childhood Cancer Data Initiative in 2019, a significant USD 50 million initiative aimed at facilitating data sharing. This initiative enables the aggregation of pediatric patient-level data from across the country and, in some cases, globally. This has the potential to greatly expedite scientific discovery in various fields of oncology. The development of extensive, collaborative data repositories in pediatric cancer is still in its early phases. However, there are already numerous smaller patient registries that focus on specific diseases, societies, or geographic regions. These registries can serve as a basis for effective data sharing and exploration [163]. There are several data-sharing initiatives in place, such as pediatric cancer data, which are backed by various sub-discipline interest groups, national health agencies, and academic consortia. Table 2 provides a summary of important data sources and initiatives based in the United States [163]. The National Cancer Institute’s Surveillance, Epidemiology, and End Results (SEER) Program is a registry that gathers information on cancer incidence and survival from 19 US geographic areas, which together account for about 34% of the country’s population. Research on pediatric cancers has made great use of SEER, which contains data on all ages [164].

It is significant to comprehend the pathology of childhood cancers at the genomic and epigenetic levels. Pediatric Cancer Genomic Data at St. Jude Cloud provides access to a vast amount of pediatric cancer genomic data (totaling 1.25 petabytes and freely available), including 12,104 whole-genome, 7697 whole-exome, and 2202 transcriptome data, as well as clinical data from St. Jude Children’s Research Hospital. This resource is expanding rapidly with regular data uploads from St. Jude’s prospective clinical genomics programs, and it also allows researchers worldwide to explore and analyze these data to gain insights into the genetic basis of pediatric cancers and ultimately develop better treatments and therapies [165]. More robust repositories of genomic data for pediatric cancer are crucial for conducting research in this field. Furthermore, it is essential to gather information on the adverse events (AEs) associated with chemoradiotherapy and targeted therapy to evaluate the efficacy and safety of pediatric cancer treatment. Hence, it is crucial to establish a genomic database of childhood cancer data to facilitate research in this field [165].

## 5. Clinical Implications and Genetic Counseling

The clinical interpretation of genetic variants associated with cancer predisposition syndromes is challenging due to limited data evaluation resources. Even though WGS/WES produces enormous sequencing information on pediatric cancer samples, more ClinVar and clinical genetics database resources about all the predisposition variants are required to classify and interpret the variants. Currently, there is a lack of knowledge in identifying and interpreting the genetic variation in cancer predisposition genes and due to this reason, many genetic variants are presently classified as VUSs [166]. The interpretation of CPS genetic variants will inevitably vary between laboratories depending on the software and databases used [167]. A database comprising CPS variants should be developed to decrease VUSs and improve the population-specific knowledge of variants. Insufficient resources on CPS genetic variants make it difficult to classify variants found in pediatric cancers using NGS technologies, leading to conflicting interpretations and increased challenges in reliable interpretation. Despite sequencing a large number of samples, many numbers of genetic variants in genomic NBS have remained of uncertain significance (VUS) [51].

High throughput sequencing in pediatric cancer patients has uncovered a surprisingly high prevalence (8–18%) of pathogenic and likely pathogenic germline variants in CPS genes, which varies depending on the type of tumor and stage of the disease [3,12,61,76,81,168,169]. The interpretation of the CPS screening results remains challenging since not all genes are known to be associated with pediatric cancer or the patient’s tumor entity. However, identifying an underlying pathologic or likely pathologic (P/LP) germline variant is crucial for patients, as it can potentially decrease morbidity and mortality rates for both patients and their family members. Possible outcomes may involve adjustments to the treatment plan, participation in monitoring programs, and/or predictive testing for family members.

In general, genetic counseling for families with pediatric CPSs should follow best practices to ensure appropriate management and support [170]. It is important to have a multidisciplinary approach involving pediatricians, oncologists, geneticists, and psychologists to provide comprehensive care. The early recognition of genetic predispositions is crucial for tailored treatment and specific surveillance for syndrome-related malignancies [171]. It is imperative that genetic counseling incorporates the recognition of symptoms that could potentially signify the existence of hereditary cancer predisposition syndromes [172]. A comprehensive history and examination, appropriate genetic testing, and early consultation with a clinical geneticist should all be components of the counseling process. The prognosis of cancer predisposition syndrome carries significant implications for both patients and their families, encompassing the potential implementation of risk-reducing interventions, targeted treatment approaches, and surveillance systems. Furthermore, genetic counseling enables individuals to investigate alternative reproductive methods to guarantee the absence of any adverse effects on subsequent generations [173].

On the contrary, genetic counseling for families with pediatric CPSs encounters several challenges. A major challenge pertains to the underdiagnosis of these syndromes among pediatric patients, a circumstance that may impede prompt detection and suitable treatment. Another challenge is the need for a multidisciplinary team with specific training to provide comprehensive care for these conditions. Clinicians must contemplate a multitude of factors, such as the histopathological subtype of the tumor, family and patient medical history, and specific clinical symptoms, to suspect the presence of a predisposing condition due to the intricate nature of these syndromes. Further complicating the diagnosis and treatment of these syndromes is the heterogeneity of genetic alterations observed in pediatric malignancies, which includes somatic and germline mutations. Obstacles to genetic assessment referrals include inadequate family history knowledge, inconsistent pedigree elicitation, and the prioritization of urgent care requirements. To surmount such challenges, it is imperative to educate healthcare providers, establish unambiguous referral protocols, and foster cooperation with genetic counselors to guarantee accurate evaluation and control of pediatric cancer predisposition syndromes [174].

## 6. Cost-Effectiveness of Genomic Newborn Screening for CPS

Many challenges arise when examining the cost-effectiveness of genomic screening of newborns for CPSs [6]. The extensive range of hereditary disorders that may be unveiled via genome sequencing, in addition to the subsequent ramifications and corresponding interventions, pose a formidable challenge in formulating an economic framework that adequately encompasses the effects of genome sequencing [6]. The inclusion of both patient and physician preferences in the economic model, considering the role of physicians as gatekeepers for patients, introduces an additional level of complexity to the discussion [175]. An effective strategy could involve prioritizing the cost-effectiveness of secondary genetic findings as a starting point [176].

There is limited research on the cost-effectiveness of genomic sequencing technologies for childhood cancer screening in the clinical setting, yet an evaluation of the traditional diagnostic approach suggests that incorporating NGS during the initial clinical visit would result in substantial cost savings. Recent analyses of critically ill infants diagnosed with rapid whole genome sequencing (rWGS) have identified sizeable net cost savings and reductions in hospital length-of-stay [177]. The study conducted by Tak et al. recently illustrated the economic and cost-effectiveness of early cancer detection in infants afflicted with Li–Fraumeni syndrome [178]. Many studies reported that rWGS in NGS brings cost-effectiveness in diagnosing suspected single-locus genetic disorders for newborns. Employing genome sequencing in clinical healthcare settings for screening pediatric CPSs brings considerable cost savings.

In general, the cost of newborn genomic screening can vary significantly depending on several factors, including the specific tests included in the screening panel, the technology used for sequencing, the laboratory conducting the testing, and any additional services provided, such as genetic counseling and interpretation of results [179]. In high-income countries where newborn genomic screening is available, the cost can range from a few hundred dollars per infant (approx. 600 USD cost for newborn genome screening for all the conditions) [180]. This cost may cover the sequencing itself, as well as interpretation of the results, genetic counseling, and follow-up testing if necessary [180].

Traditional methods for screening pediatric cancers may include various diagnostic tests such as imaging studies (X-rays, CT scans, MRI), blood tests, biopsies, and bone marrow aspiration. The cost of these traditional screening/surveillance methods can also vary depending on the specific tests performed, the frequency of testing, and the healthcare system or country. The cost of screening CPSs through traditional methods is estimated to be around ~2200 USD [66].

Compared to traditional methods, newborn genomic screening has the potential to identify genetic predispositions to certain pediatric cancers at an earlier stage, allowing for earlier intervention and potentially better outcomes [181,182,183]. It is important to note that the cost of newborn genomic screening may decrease over time as technology advances and becomes more widely adopted [184].

## 7. Integration of Pediatric CPS Screening in the Healthcare System

Precision Public Health is a global public health initiative to deliver the right intervention at the right time to the right population to safeguard population health using genomic screening of newborns for CPSs. A research study by Chen et al. highlights the immense value of genome sequencing in NBS, representing a substantial advancement toward achieving precision public health in China [39]. However, there are a few intricacies and factors that need to be carefully addressed before the integration of genomic newborn screening for pediatric CPSs into routine healthcare services [14].

Although NBS is widely recognized as a significant accomplishment in public health, it also has negative consequences, such as medicalizing families who receive a false positive result or subjecting children with a minor or uncertain condition to excessive treatment [185]. Information, care, support, monitoring, and management must be prudently planned to enhance the outcome for patients and their families and to keep these negative impacts from outweighing the positive ones. European Reference Networks (ERNs), the International Society for Neonatal Screening (ISNS), and the European Society for Immunodeficiencies (ESID) have devised important recommendations or guiding principles for effective newborn genome screening programs [186]. These are summarized below.

1. The selection of new conditions in NBS panels should rely on established criteria that have been published. The methods should be standardized, transparent, and open to public examination. Furthermore, the outcomes of the debates should be made available to the public.
2. Parents should have access to information, ideally provided during pregnancy, that explains the diseases that will be tested for and the consequences of receiving a positive result. This will enable them to make an informed decision about whether to participate.
3. Precise definitions of the illnesses being examined should be established before planning the screening process.
4. Laboratories that have accreditation demonstrating compliance with international standards for laboratory performance should be chosen for screening.
5. Key performance indicators (KPIs) about the complete NBS process—including blood sampling, transport conditions, blood spot quality, time to generate laboratory results, and referral of screen-positive cases should be measurable by laboratories and programs.
6. Parents should have access to information when they are sent for clinical assistance. Their initial contact should be with a knowledgeable doctor who can provide support. If necessary, genetic counseling should also be offered.
7. It is important to implement and consistently follow confirmatory testing with a brief and clearly defined time frame to alleviate parental anxiety and tension.
8. Strategies for evaluating long-term outcome data ought to be established and duly documented.
9. Adverse screening results should be duly informed to all parents and documented in the child’s medical record.
10. Practices should be reviewed, and policies for accessing and storing residual blood spot samples should be established.

A priori we acknowledge differences between high-income and developing countries in their abilities to implement gNBS programs for pediatric cancers. The constraint is not only related to resources but also to the type of healthcare system providing clinical services (such as centralized vs. decentralized system) and the extent of medical or healthcare insurance coverage rules or policies. In high-income countries, newborn screening is typically covered by medical insurance or government-funded healthcare systems [187]. It is important to note that the specific tests included in newborn screening panels may vary from nation to nation [188]. However, regardless of the specific tests performed, newborn screening is generally considered a standard and essential component of pediatric healthcare in high-income countries [187].

In developing countries, gNBS is not widely implemented yet as a national program since it carries a significant financial burden. However, they contribute to more than half of the total births globally [189]. Since the cost of an NBS program competes with the country’s other health issues, this is usually compounded by numerous obstacles like a poor economy, a lack of government support, partial insurance coverage, inadequate public health education, a lack of awareness among healthcare workers, and early discharge from hospital. Integrating NBS with the national healthcare/insurance system is indispensable for successful implementation in developing countries [189].

## 8. Challenges and Limitations

Genome sequencing has been used extensively in clinical applications, and its implementation in NBS for CPSs intensifies many challenges and raises profound, deep societal concerns on the principles of nonmaleficence, autonomy, beneficence, and the safeguarding of each child’s potential future [190,191,192]. The integration of genome sequencing of newborns for CPSs into routine neonatal screening as a standard healthcare service presents a distinct array of challenges. Further research is required to elucidate the intricacies of the numerous genetic variants associated with CPSs and their manifestations, factors that may contribute to results that are ambiguous or deceptive. For the pathogenic variants in cancer predisposition genes over which adequate knowledge prevails, genomic data interpretation is a complex task that demands significant expertise. One limitation is the low specificity of clinical checklists, which can be missing a significant number of children with genetic predisposition. Another limitation is the inclusion of genes in CPS gene panels that have not been strongly linked to childhood cancer predisposition yields both false-positive and false-negative outcomes [131]. Furthermore, the reliance on family history and phenotypic features for screening may not always accurately identify children with cancer predisposition syndromes, especially with the increasing identification of de novo germline mutations. These outcomes, in turn, can induce unwarranted distress, necessitate further examinations, or overlook crucial diagnoses. These limitations highlight the need for improved screening criteria that consider the specific genetic and clinical characteristics of childhood cancer predisposition syndromes [151].

Ethical and legal considerations arise when dealing with genomic data, especially in the case of newborns who are unable to give informed consent. Issues related to data privacy, data security, accessibility, and the possibility of discrimination based on genomic information must be resolved. Implementing genome sequencing as a regular part of newborn screening would necessitate significant investment in infrastructure and resources [41]. This would include having trained professionals who can conduct the tests, analyze the findings, and offer genetic counseling. The major challenge lies in standardizing sequencing methods, data interpretation, reporting results and ensuring quality control across various laboratories and platforms [6]. Efficient and comprehensive systems are necessary to provide ongoing support and care for individuals who have been diagnosed with genetic conditions through NBS. Further research is needed to better understand the psychological and social effects of genomic information on families, especially when it pertains to the risk of conditions that may only appear later in life. Ensuring equal access to genome sequencing in NBS poses a significant challenge. There is a growing concern regarding the potential unequal access to the benefits of genome sequencing, which could further widen health disparities based on income or geographic location [193].

Since public health programs have limited funding, customized and specific gene panels are required for screening CPS conditions in newborns. In the past fifteen years, the price of genome sequencing has decreased dramatically, and this trend is expected to continue due to ongoing technological developments. Advanced research studies are required to establish the clinical efficacy and cost-effectiveness of sequencing large gene panels (which includes detection of structural variation and copy number variation) for newborn screening at the population level. Additionally, unresolved health policy and ethical concerns must be resolved before the implementation of such panels for newborn screening within a given jurisdiction. Also, these gene panels should be validated at the clinical level for CPSs, so that the specificity and sensitivity of genome sequencing technology and analytical process will be highly reproducible to incorporate into population-wide newborn screening programs.

## 9. Precision Medicine Approaches for Risk Stratification

Large-scale molecular profiling, next-generation sequencing, and whole-exome sequencing are examples of precision medicine techniques for risk stratification of pediatric cancer predisposition syndromes [194]. These methods have been applied to detect germline mutations in cancer susceptibility genes and somatic tumor modifications, yielding important data for therapeutic choices. Furthermore, risk stratification for pediatric cancer predisposition syndromes has been enhanced by the introduction of innovative diagnostic techniques, such as the International Prognostic Scoring System-Molecular (IPSS-M), which considers hematologic characteristics, cytogenetic abnormalities, and somatic gene mutations [195,196]. These advancements in precision medicine have the potential to improve outcomes and minimize side effects in pediatric oncology.

Several large international programs, such as the MAPPYACTS study [197], the INFORM registry [198], the Zero Cancer Childhood Initiative (ZERO) [199], the NCI-COG Pediatric MATCH trial (PedMATCH) [200], and the SickKids Cancer Sequencing Program (KiCS) [201], have highlighted the importance of tumor biopsy and genetic profiling programs in biomarker-driven, stratified medicine trials for high-risk or relapsed cancers in children and adolescents.

## 10. Longitudinal Studies for Outcome Assessment

Research indicates that the ongoing management of persons identified through newborn screening, along with the continuous collection of health information, are critical foundations for an effective newborn screening system in clinical settings. The Newborn Screening Translational Research Network (NBSTRN) has established a consensus-driven approach involving clinical care specialists to produce, maintain, and enhance sets of questions and answers grouped into common data elements (CDEs) [202]. This endeavor led to the identification of key data components for the long-term follow-up (LTFU) initiatives carried out by state NBS programs. Researchers, advocates, policymakers, and physicians can utilize these data elements to assess the health outcomes of newborns identified by NBS. The Longitudinal Pediatric Data Resource (LPDR) may benefit from the inclusion of genotype–phenotype data linked to pediatric cancer-predisposing syndrome by the NBSTRN [203]. This will improve knowledge of pediatric cancers and their implications for early detection and treatment. The creation of the LPDR for the collection, exchange, and analysis of longitudinal data promotes research and makes it easier to apply findings to therapeutic settings [204].

Studies suggest that the continual supervision of individuals identified through newborn screening, together with the consistent gathering of health data, are essential pillars for a successful newborn screening system in clinical environments. The NBSTRN has implemented a collaborative method, incorporating professionals in clinical care, to create, manage, and improve collections of questions and answers organized into standardized CDEs [203]. This undertaking resulted in the identification of essential data elements for the implementation of LTFU activities conducted by state NBS programs. Researchers, advocates, policymakers, and physicians can use these data items to evaluate the health results of babies identified by NBS. The LPDR could be enhanced by incorporating genotype–phenotype data associated with pediatric cancer-predisposing syndrome, as provided by the NBSTRN. This initiative aims to enhance understanding of pediatric malignancies and their significance in terms of early identification and therapy. The establishment of the LPDR facilitates the gathering, sharing, and examination of longitudinal data, hence enhancing research and streamlining the application of findings in therapeutic contexts [203].

The LPDR has been employed in several endeavors, including a decade-long initiative to gather, scrutinize, and distribute health data on individuals with different medical conditions. As the data sets in the LPDR expand, it will be advantageous for the NBS community to use this information to enhance the healthcare and management of newborns with a cancer predisposition condition through NBS, inform policy and funding decisions, evaluate the effectiveness of NBS, and deepen our knowledge of these pediatric cancers [203].

## 11. Future Prospects for Pediatric Cancer Screening Programs

In general, germline gene mutations in CPS have high/intermediate risks of cancer. Further opportunities to use cancer screening programs as a preventive healthcare system to improve the management of pediatric cancer patients should be vigorously pursued. Other genomic architectural components of CPSs are also important, and the interplay of various genetic variants will reveal clinically relevant insights [205]. Various post-zygotic events that contribute to cancer predisposition have been discovered. For instance, children with bilateral Wilms tumor often exhibit H19 hypermethylation [206]. Identifying de novo germline mutations (cancer-causing) through pediatric cancer screening programs (using gNBS) will bring personalized care for newborns with a family history of malignant tumors. Pediatric cancer screening programs would be greatly facilitated by data-sharing initiatives from pediatric cancer networks and registries of mutation carriers. The inclusion of germline CPG testing in clinical trials and improved collaboration between somatic and germline cancer research will greatly enhance the values of this program. Improving the accessibility of cancer predisposition genetic (CPG) testing for pediatric cancer patients is crucial for ensuring timely diagnoses, personalized treatment, and informed decision-making. More advancements in sequencing technologies will bring efficient analysis and interpretation of CPGs on a large scale.

Advancements in medical research and technology continue to improve the early detection of pediatric cancers. The early detection of genetic predispositions can lead to personalized screening programs and interventions tailored to each child’s unique risk profile. Artificial intelligence (AI) and machine learning (ML) algorithms can be leveraged to analyze gNBS data from pediatric cancer patients to identify patient-specific genetic variants susceptible to CPS that may be missed by human observers. Public health initiatives and awareness campaigns play a vital role in promoting pediatric cancer screening and diagnosis. By educating healthcare providers, parents, and caregivers about the importance of screenings and recognizing the signs and symptoms of childhood cancer, we can ensure timely diagnosis and treatment of newborns who are genetically susceptible to childhood cancer. Overall, the future of pediatric cancer screening programs looks promising, with ongoing advancements in technology, research, and collaborative efforts aimed at improving early detection and better treatment outcomes for pediatric malignancies.

## 12. Conclusions

The population-wide genome screening of newborns may significantly decrease mortality associated with childhood cancers. Employing genome sequencing in pediatric cancer diagnosis, including a wide spectrum of CPSs, could potentially be cost-effective since sequencing costs are decreasing dramatically. Prior to implementing population-based newborn screening tests, there is a need for clinical studies investigating crucial factors such as parental uptake of testing, the impact of a genetic CPS diagnosis on families, adherence to surveillance, and the effectiveness of surveillance in preventing advanced disease.

The gNBS program is a successful public healthcare initiative, and it is recommended with applicable standard guidelines and ethical considerations. Its effectiveness and accessibility can strengthen the public’s confidence in NBS programs. However, integrating WGS into NBS could present new challenges impacting its inclusivity, credibility, and public trust. Healthcare professionals (HCPs) may encounter ethical and practical challenges during clinical practice. Clinical standards and guidelines must be revised for the responsible and ethical integration of WGS-NBS. Clinical healthcare organizations should investigate parental consent before screening the newborn for CPS, familial history of a genetic syndrome, and the impact of diagnosing genetic CPS on families before this testing can be proposed as a component of population-based newborn screening for CPS.

Considering the multiple genetic changes that cause CPS and the surprising connections between genotype and phenotype, it is recommended to have a large set of genes for the CPS screening panel. Both clinical screening and newborn genome testing would be conducted at the beginning of cancer patient care. The results could have immediate implications for patient surveillance, and, in certain cases, they assist in the early treatment of CPS. In addition to implementing suitable surveillance measures, it is critical to offer children and their families referrals to psychosocial support and genetic counseling. Testing long-term survivors could introduce a significant bias due to the early mortality of patients with CPSs. Additional research is required to ascertain the practicality and precision of this method in broader cohorts of pediatric cancer patients.

In conclusion, targeted newborn genomic screening has the potential to identify germline cancer predisposition in neonates, who have a high risk of developing CPS malignancy before 20 years of age. It allows increased opportunities for cancer surveillance and intervention for newborns as compared to traditional newborn testing approaches. In this review article, we highlight solutions to challenges toward the goal of minimizing harm and maximizing the substantial potential benefits of gNBS for CPSs.

## Figures and Tables

**Table 2:** Pediatric cancer data sources and data sharing initiatives from the United States and European Union.

Data Sharing Initiative	Organization	Data Domain	Data Source Description	Website
Cancer Research Data Commons (TARGET)	National Cancer Institute (US NCI)	Pediatric Oncology (Medical registries)	Therapeutically Applicable Research to Generate Effective Treatments (TARGET) consortium for clinical trials in childhood and adolescent cancer research	https://www.cancer.gov/research/nci-role/bioinformatics/cancer-research-data-ecosystem-infographic (accessed on 10 March 2024)
Cancer Research Data Commons (Genomic Data Commons)	National Cancer Institute (US NCI)	Pediatric oncology (Clinical Genomics)	To harmonize NCI’s cancer genomics data, including processing clinical genomics data also developing data models for clinical data and biospecimens	https://gdc.cancer.gov/ (accessed on 10 March 2024)
Childhood Cancer Research Network (EveryChild)	Children’s Oncology Group (COG)	Pediatric oncology (Clinical data registries)	Medical registries of pediatric cancer data registered with Children’s Oncology Group (COG)	http://projecteverychild.org/ (accessed on 7 March 2024)
Kids First Data Resource Center (DRC)	Gabriella Miller Kids First Data Resource Center (Kids First DRC) program	Pediatric oncology (Clinical genomics)	Six partner studies, institutions, and consortia. Kids first data are functionally equivalent to other extensive genomic efforts such as Genotype-Tissue Expression (GTeX) and NCI Genomic Data Commons	https://kidsfirstdrc.org (accessed on 7 March 2024)
European Cancer Information System	Knowledge Centre on Cancer (European Commission)	Pediatric Oncology (Demographics of Europe)	ECIS brings data together from Europe to better monitor trends and outcomes for different diagnostic groups of childhood cancer	https://ecis.jrc.ec.europa.eu/index.php (accessed on 10 April 2024)
National Program of Cancer Registries (Pediatric and Young Adult Early Case Capture Program)	Centers for Disease Control (US CDC)	Pediatric Oncology (Clinical data registries)	Cancer registries of US population within 30 days of diagnosis	https://www.cdc.gov/cancer/npcr/index.htm (accessed on 21 March 2024)
NCI-COG Pediatric MATCH (Molecular Analysis for Therapy Choice)	National Cancer Institute and Children’s Oncology Group	Pediatric Oncology (Clinical genomics data registries)	Precision medicine program for pediatric cancer patients using clinical data from COG consortium	https://www.cancer.gov/about-cancer/treatment/clinical-trials/nci-supported/pediatric-match (accessed on 21 March 2024)
PeCan (St. Jude Cloud)	St. Jude’s Hospital	Pediatric Oncology (Clinical Genomics)	Curated data from ~900 pediatric cancer samples at St. Jude and collaborators	https://pecan.stjude.cloud/ (accessed on 21 March 2024)
Pediatric Cancer Data Commons (PCDC)	University of Chicago and partners	Pediatric oncology (Clinical data registries)	• International Neuroblastoma Risk Group—INRG), International Soft-Tissue Sarcoma Consortium—INSTRuCT, Malignant Germ Cell International Consortium—MaGIC, and acute myelogenous leukemia • TARGET • Nationwide Children’s Biopathology Center	http://commons.cri.uchicago.edu (accessed on 11 March 2024)
St. Jude CARES	St Jude’s Hospital	Pediatric oncology (Clinical data registries)	Hospital-based pediatric cancer registration system for low- and middle-income countries	https://www.stjude.org/global/sjcares/registry.html (accessed on 11 March 2024)
St. Jude Cloud	St. Jude’s Hospital	Pediatric Oncology (Clinical Genomics)	Genomics data from pediatric cancer patients diagnosed and treated at St. Jude	https://www.stjude.cloud/ (accessed on 11 March 2024)
Treehouse Childhood Cancer Initiative	University of California, Santa Cruz	Pediatric Oncology (Clinical Genomics)	Publicly available gene expression data from >10 K pediatric tumor samples by combined efforts of nine hospitals and consortia	https://treehousegenomics.soe.ucsc.edu/explore-our-data/ (accessed on 18 March 2024)
UNderstand CANcer (UNCAN.eu)	European Union	Pediatric Oncology (Cancer Research)	UNCAN.eu is an initiative from European Union to support the creation of a sustainable platform to connect cancer research across member states	https://uncan.eu/ (accessed on 10 April 2024)
